# Comparison of Various Surgical Approaches for Moderate-to-Severe Ischemic Mitral Regurgitation: A Systematic Review and Network Meta-Analysis

**DOI:** 10.31083/j.rcm2511425

**Published:** 2024-11-25

**Authors:** Zhili Wei, Shuai Dong, Xuhua Li, Yang Chen, Shidong Liu, Bing Song

**Affiliations:** ^1^The First Clinical Medical College, Lanzhou University, 730000 Lanzhou, Gansu, China; ^2^Department of Cardiovascular Surgery, First Hospital of Lanzhou University, 730000 Lanzhou, Gansu, China

**Keywords:** coronary artery bypass grafting, mitral valve repair, mitral valve replacement, ischemic mitral regurgitation, systematic review/network meta-analysis

## Abstract

**Background:**

This study aims to systematically review the efficacy of various surgical approaches in the treatment of ischemic mitral regurgitation (IMR).

**Methods:**

A comprehensive literature search was conducted using computerized databases, including PubMed, Cochrane Library, Embase, and Web of Science, up to February 2024. In our network meta-analysis, we utilized the Cochrane Handbook tool for quality evaluation, while a consistency model and the odds ratio (OR) were used to compile and analyze the data from the studies included, employing Stata 17.0 software for this purpose.

**Results:**

The systematic review included a total of 20 randomized controlled trials (RCTs), which collectively involved 3111 patients and evaluated six different surgical techniques. The network meta-analysis demonstrated that mitral valve repair (MVr) exhibited a significant reduction in 30-day all-cause mortality rates when compared to coronary artery bypass grafting (CABG), mitral valve replacement (MVR), CABG combined with MVR, and transcatheter mitral valve edge-to-edge repair (TEER) using MitraClip. Furthermore, probability ranking analysis suggested that MVr may be the most effective approach in reducing 30-day all-cause mortality, while CABG combined with MVr had significantly fewer renal complications compared to CABG combined with MVR. Probability rankings also indicated that CABG+MVr may be the most effective technique in minimizing renal complications. However, there were no statistically significant differences observed in other outcome measures among the different surgical techniques.

**Conclusions:**

Current limited evidence indicates that CABG combined with MVr may be the best surgical approach for patients with IMR. However, these conclusions are tentative and require further confirmation from more additional high-quality studies.

**INPLASY Registration Number:**

INPLASY202420049. This study can be accessed at the following detailed address: https://inplasy.com/inplasy-2024-2-0049/, last accessed on February 11, 2024.

## 1. Introduction

Ischemic mitral regurgitation (IMR) is a prevalent complication in post-myocardial infarction patients, with an estimated prevalence of approximately 40%–50%, and nearly 10% of these patients experiencing moderate to severe IMR [1, 2]. This condition is associated with an increased risk of mortality and heart failure, with the one-year mortality rate estimated at 10% for mild IMR, rising to 40% for severe IMR. Moreover, patients with myocardial infarction and concurrent IMR face a threefold increased risk of developing heart failure [3]. The primary mechanisms for IMR following myocardial infarction are identified as follows: (1) Partial obstruction or blockage of the coronary artery resulting in myocardial ischemic necrosis, subsequent rupture of papillary muscles and chordae tendineae, and consequent valve prolapse and IMR; (2) Ventricular remodeling induced by coronary heart disease, characterized by ventricular dilation resulting in passive enlargement of the mitral annulus and relative insufficiency of mitral closure. Displacement of papillary muscles and reduced annulus closure strength also contribute to IMR [4, 5]. IMR is characterized by localized wall motion abnormalities, often secondary to coronary artery disease, with the mitral leaflets and chordae tendineae typically less structurally affected [6, 7].

Currently, IMR is primarily managed through a variety of treatment modalities including medications, cardiac resynchronization therapy, coronary artery bypass grafting (CABG), mitral valve repair (MVr), mitral valve replacement (MVR), interventional treatments, or a combination of these surgical approaches. Some perspectives suggest that CABG effectively restores myocardial blood flow, ameliorates ventricular remodeling, and reduces wall motion abnormalities [8, 9]. However, reports suggest that CABG alone in patients with moderate to severe IMR may result in a mortality or heart failure rate up to 50% [10, 11]. Moreover, the efficacy of CABG alone may be temporary, with recurrence of mitral regurgitation and other complications frequently observed postoperatively [12]. Persistent mitral regurgitation increases ventricular load, potentially leading to eventual heart failure [13]. While MVR or MVr alone can effectively address mitral regurgitation, they do not address ischemic myocardium. Post-MVR, long-term anticoagulation is required, with an annual incidence of bleeding or thromboembolism ranging from 2% to 7%, along with risks of paravalvular leak and endocarditis [9]. Combining CABG with MVr or MVR considerably extends surgery duration, extracorporeal circulation, intubation, and anesthesia times, thereby increasing the risk of complications [14]. Pharmacotherapy and cardiac resynchronization therapy are primarily utilized for mild IMR. The surgical treatment of IMR remains a subject of debate, prompting this study to employ network Meta-analysis to evaluate the efficacy of various surgical interventions for IMR, with the aim of offering guidance for clinical decision-making.

## 2. Methods

A systematic review of selected publications has been conducted following the preferred reporting items for Systematic Reviews and Meta-analysis guidelines, and the full research protocol has been registered in the INPLASY database (INPLASY202420049).

### 2.1 Search Strategy

A comprehensive search was performed in PubMed, Embase, Cochrane Library and Web of Science databases, covering the period from their establishment to February 2024. In addition, manual searches of relevant conference proceedings were conducted to enhance the literature review. The search strategy employed a combination of Medical Subject Headings (MeSH) terms and free-text keywords, focusing on terms such as ischemic mitral valve incompetence, ischemic mitral valve insufficiency, ischemic mitral valve regurgitation, ischemic bicuspid heart valve incompetence, mitral valve replacement, coronary artery bypass graft, edge-to-edge transcatheter mitral valve repair, and mitral valve transcatheter edge-to-edge repair.

### 2.2 Inclusion and Exclusion Criteria

The inclusion criteria for studies were as follows: (1) Randomized controlled trials (RCTs). (2) Patients diagnosed with moderate-to-severe IMR using cardiac color Doppler ultrasound [15]. (3) Interventional methods comprising CABG, MVR, MVr, transcatheter mitral valve edge-to-edge repair (TEER) with the MitraClip device (Abbott, Santa Clara, CA, USA), CABG+MVR, and CABG+MVr. (4) Study outcomes covering 30-day all-cause mortality; major bleeding events; stroke; renal complications; neurological complications; respiratory complications. Exclusion criteria included: (1) Repeatedly published studies. (2) Literature with inaccessible full texts or incomplete data. (3) Reviews, conference abstracts, or correspondence. (4) Non-clinical research such as animal studies.

### 2.3 Literature Selection and Data Extraction

The literature selection and data extraction were conducted independently by two researchers, with a process for cross-verification in place. Disagreements were resolved through mutual discussion or consultation with a third party. The extracted data included: (1) Baseline data of the studies: first author, publication year, country, study type, intervention actions, and sample size. (2) Characteristics of patients: sex, age, body mass index (BMI), hypertension, hyperlipidemia, diabetes, history of myocardial infarction, etc. (3) Data required for bias risk evaluation. (4) Data on outcome measures: 30-day all-cause mortality; major bleeding events; stroke; renal complications; neurological complications and respiratory complications.

### 2.4 Bias Risk Assessment of Included Studies

The quality assessment of the included RCT studies was independently conducted by two researchers using RevMan 5.3 software (Cochrane Collaboration, London, UK), a tool recommended by the Cochrane Collaboration for systematic reviews. This tool evaluates the risk of bias across six aspects: method of randomization, allocation concealment, blinding, completeness of outcome data, publication bias, and other biases. The findings were then cross-checked to ensure consistency and accuracy in the assessment of bias risk.

### 2.5 Statistical Analysis

Network meta-analysis in this study was conducted using Stata 17.0 software (Stata Corp LLC, College Station, TX, USA). The effect size for count data was determined using the odds ratio (OR), and for measurement data, the mean difference (MD) was used. Both effect sizes were accompanied by their respective 95% confidence intervals (CI). Heterogeneity among the results of the included studies was assessed using the Chi-squared test (α= 0.1) combined with *I*^2^ quantitative analysis. This network meta-analysis was based on a frequentist approach, initially creating network diagrams to illustrate the relationships between various interventions. Consistency analysis was then conducted to assess differences between direct and indirect evidence, with inconsistency considered present if *p* < 0.05. Node-splitting methods were employed to investigate the sources of inconsistency at specific points. In the absence of inconsistency, the analysis proceeded accordingly. Furthermore, the surface under the cumulative ranking curve (SUCRA) was utilized to rank the effectiveness of different interventions, with higher SUCRA values indicating a greater impact on the outcome measure.

## 3. Results

### 3.1 Literature Search Results

**Fig. 1.**
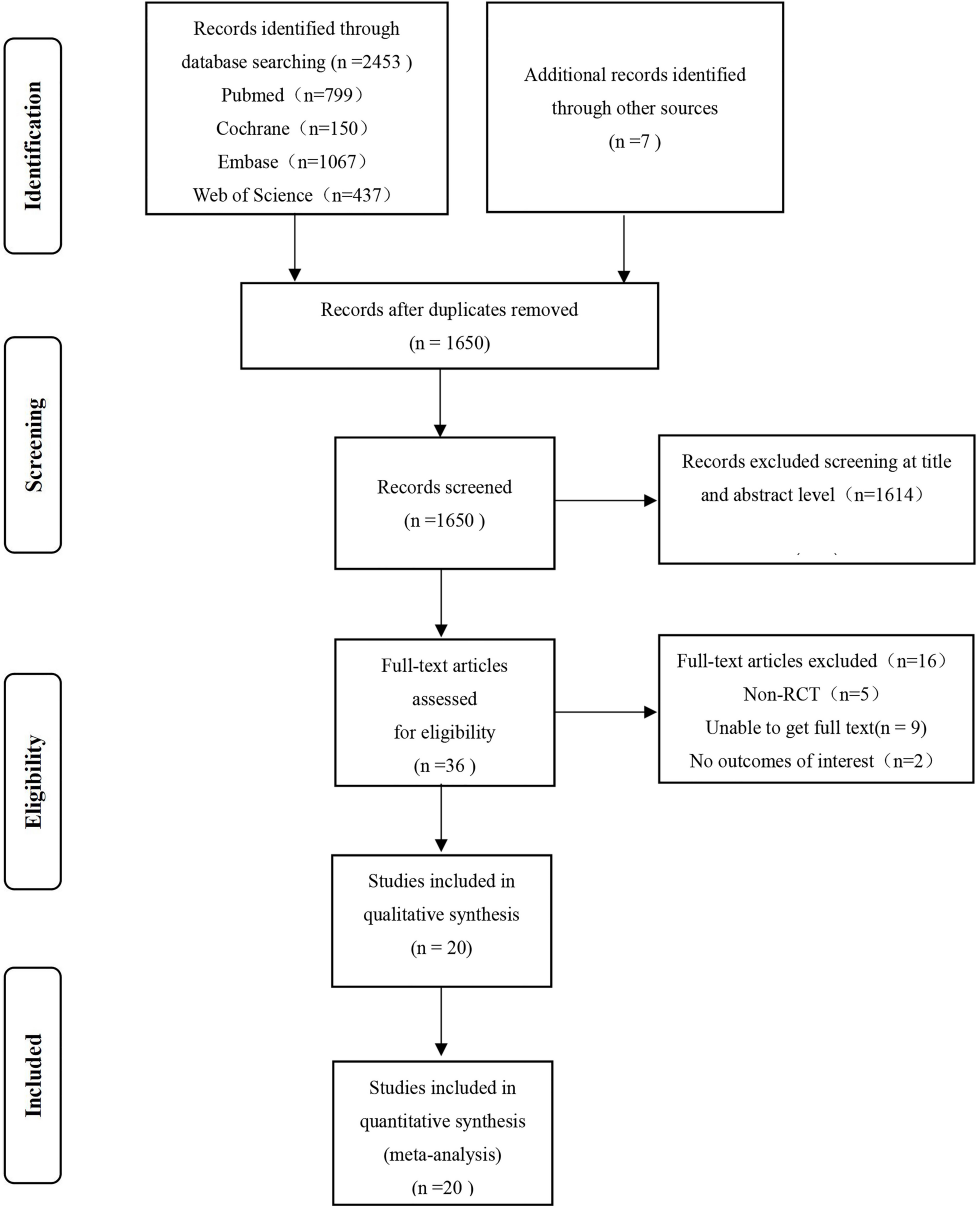
**Flow chart of the publication selection process, based on the PRISMA statement**. PRISMA, Preferred Reporting Items for Systematic reviews and Meta-Analyses; RCT, randomized controlled trial.

The initial search identified 2460 publications, of which 810 duplicates were subsequently removed. Screening of titles and abstracts led to the exclusion of 1614 publications. After a full-text review, an additional 16 publications were excluded, leaving 20 studies [16, 17, 18, 19, 20, 21, 22, 23, 24, 25, 26, 27, 28, 29, 30, 31, 32, 33, 34, 35] for inclusion. The process of literature selection is illustrated in Fig. 1.

### 3.2 Characteristics of Included Studies

The studies included were published between 2001 and 2022, involving a total of 3111 patients. Notably, Qiu *et al*. [30] had a sample size of 378, which is the largest among all the included studies. The interventions assessed in this study included CABG, MVR, MVr, TEER using MitraClip, CABG+MVR, and CABG+MVr. The average age of patients across the study was 65.6 years, with a range of 54.3 to 72.3 years. The proportion of male patients was 64.0% with a range of 33.1% to 100.0%. The prevalence of comorbidities among the patient population was as follows: hypertension was present in 55.0% of patients (range 10.8% to 93.8%), hyperlipidemia in 47.9% (range 24.0% to 77.1%), diabetes in 38.0% (range 15.6% to 66.7%), myocardial infarction in 61.8% (range 35.6% to 83.0%), COPD in 15.0% (range 0% to 50%), and atrial fibrillation in 15.9% (range 0% to 50%). Further details regarding the fundamental characteristics of these studies are provided in Table 1 (Ref. [16, 17, 18, 19, 20, 21, 22, 23, 24, 25, 26, 27, 28, 29, 30, 31, 32, 33, 34, 35]).

**Table 1.** **The general information of the included studies (T/C)**.

Study	General characteristics					Past complications (%)						Preoperative examination		Intervention
	Country	Type of study	Total (n)	Male (%)	Age	HTN	HPL	DM	MI	COPD	AF	MR area (cm^2^)	LVEF (%)	T/C
Sá, MPBO *et al*. 2013 [31]	Brazil	RCT	42	54/54	NR	94/85	NR	25/42	50/46	13/0	NR	NR	NR	CABG+MVR/CABG
Dufendach, K *et al*. 2020 [18]	US	RCT	358	51/33	69/70	87/87	77/78	47/49	54/58	41/27	NR	NR	49/43	CABG+MVR/CABG+MVr
Lee, M *et al*. 2018 [26]	China	RCT	22	75/100	63/61	75/44	50/28	25/67	NR	50/6	50/33	NR	46/30	CABG+MVR/CABG+MVr
Chan *et al*. 2012 [17]	US	RCT	73	33/74	70/71	59/50	NR	38/35	72/74	3/6	10/6	0.18/0.21	NR	CABG/CABG+MVr
Fattouch *et al*. 2022 [19]	Italy	RCT	102	65/63	66/64	43/54	NR	59/58	82/83	9/8	NR	NR	43/42	CABG/CABG+MVr
Goland *et al*. 2009 [21]	US	RCT	83	NR	68/69	61/65	NR	29/36	61/44	14/15	NR	NR	37/39	CABG+MVr/CABG
Jeong *et al*. 2012 [23]	Korea	RCT	140	58/73	65/64	69/54	NR	46/49	NR	NR	4/18	NR	NR	CABG/CABG+MVr
Ji *et al*. 2019 [24]	China	RCT	200	83/78	74/74	67/63	24/28	37/41	45/36	12/14	NR	0.17/0.15	43/47	CABG+MVr/CABG
Khallaf *et al*. 2020 [25]	Egypt	RCT	60	55/60	55/54	60/45	NR	60/55	65/60	NR	NR	NR	48/50	CABG+MVr/CABG
Tribak *et al*. 2018 [34]	France	RCT	55	86/81	58/57	71/34	42/34	NR	64/50	NR	14/0	NR	NR	CABG+MVr/CABG
Wang *et al*. 2022 [35]	China	RCT	162	49/48	72/72	11/10	NR	47/45	74/69	31/24	NR	NR	NR	CABG+MVr/MitraClip
Acker *et al*. 2014 [16]	US	RCT	251	61/62	69/68	NR	NR	38/33	79/70	NR	36/28	0.40/0.39	42/40	MVr/MVR
Gimpel *et al*. 2020 [20]	AU	RCT	119	72/67	71/72	61/72	66/72	30/33	NR	NR	NR	NR	NR	MVr/MVR
Grossi *et al*. 2001 [22]	US	RCT	223	64/59	NR	NR	NR	30/31	NR	NR	NR	NR	NR	MVr/MVR
Li *et al*. 2018 [27]	China	RCT	218	75/78	62/61	59/55	46/46	16/20	NR	6/6	7/5	NR	NR	MVr/MVR
Li *et al*. 2019 [28]	China	RCT	154	83/79	62/62	58/52	38/42	25/20	NR	8/4	17/8	NR	54/55	MVr/MVR
Qiu *et al*. 2017 [30]	China	RCT	378	69/63	66/66	NR	NR	NR	NR	NR	9/9	NR	50/58	MVr/MVR
Silberman *et al*. 2006 [32]	US	RCT	80	74/93/79	62/67/68	50/57/61	NR	45/57/46	NR	13/14/21	NR	NR	NR	MVr/MVR/CABG
Michler *et al*. 2016 [29]	US	RCT	301	NR	NR	NR	NR	NR	NR	NR	NR	NR	NR	CABG/CABG+MVr
Toktas *et al*. 2016 [33]	Turkey	RCT	90	48/41	61/63	NR	NR	59/59	NR	14/17	16/17	NR	NR	CABG+MVr/CABG
Total	NR	NR	3111	64/67	66/57	62/55	49/47	39/43	65/59	18/12	18/14	0.25/0.25	46/49	NR

NR, not reported; HTN, hypertension; HPL, hyperlipidemia; DM, diabetes mellitus; MI, myocardial infarction; COPD, chronic obstructive pulmonary disease; AF, atrial fibrillation; MR, mitral regurgitation; LVEF, left ventricular ejection fraction; RCT, randomized controlled trial; T/C, test group/control group; CABG, coronary artery bypass grafting; MVr, mitral valve repair; MVR, mitral valve replacement.

### 3.3 Risk of Bias Assessment

**Fig. 2.**
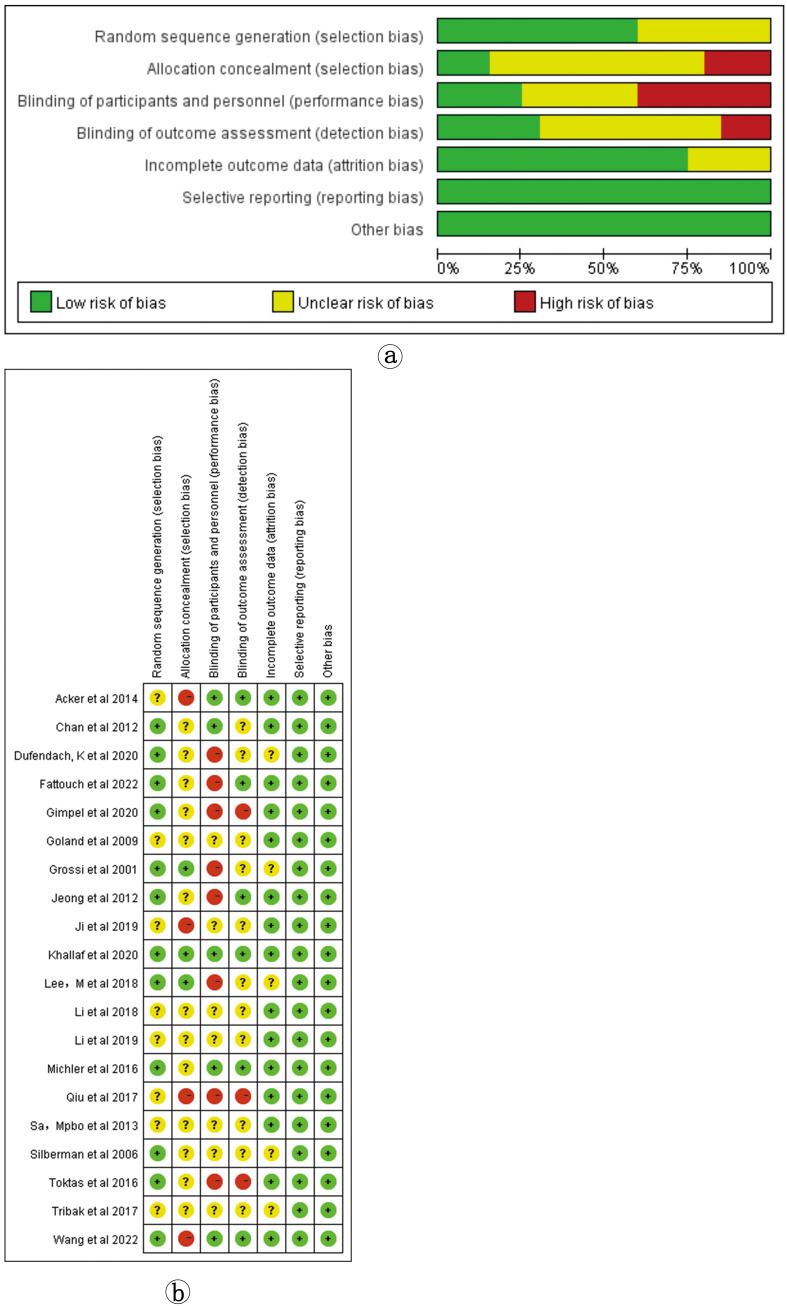
**Included study bias risk assessment results**. (a) Risk of bias bar chart; (b) Risk of bias summary chart.

The risk of bias in the included studies is shown in Fig. 2a,b.

### 3.4 Inconsistency Test

The overall consistency test revealed good alignment between direct and indirect comparisons (*p* > 0.05). Additionally, the node-splitting method was applied for local inconsistency assessment, revealing no significant inconsistency (*p* > 0.05). Consequently, a consistency model was employed for the network meta-analysis. For 30-day all-cause mortality, for example, detailed results of the inconsistency test are shown in Table 2.

**Table 2.** **30-day all-cause mortality inconsistency test**.

Intervention	Direct Coef	Std. Err	Indirect Coef	Std. Err	Difference Coef	Std. Err	*p > |z|*
CABG-CABG+MVR	–0.22	1.27	0.25	0.45	–0.47	1.35	0.73
CABG-CABG+MVr	–0.52	0.26	–1.00	1.32	0.47	1.35	0.73
CABG-MVR	–0.69	0.88	–0.30	1.63	–0.40	2.05	0.85
CABG-MVr	–1.35	0.74	–1.76	1.82	0.40	2.05	0.85
CABG+MVR-CABG+MVr	–0.77	0.36	–0.31	1.30	–0.47	1.35	0.73
CABG+MVr-MitraClip	1.22	0.68	1.65	2.97	–0.43	2.97	1.00

CABG, coronary artery bypass grafting; MVR, mitral valve replacement; MVr, mitral valve repair; Coef, coefficient; Std. Err, standard error.

### 3.5 Network Relationships

**Fig. 3.**
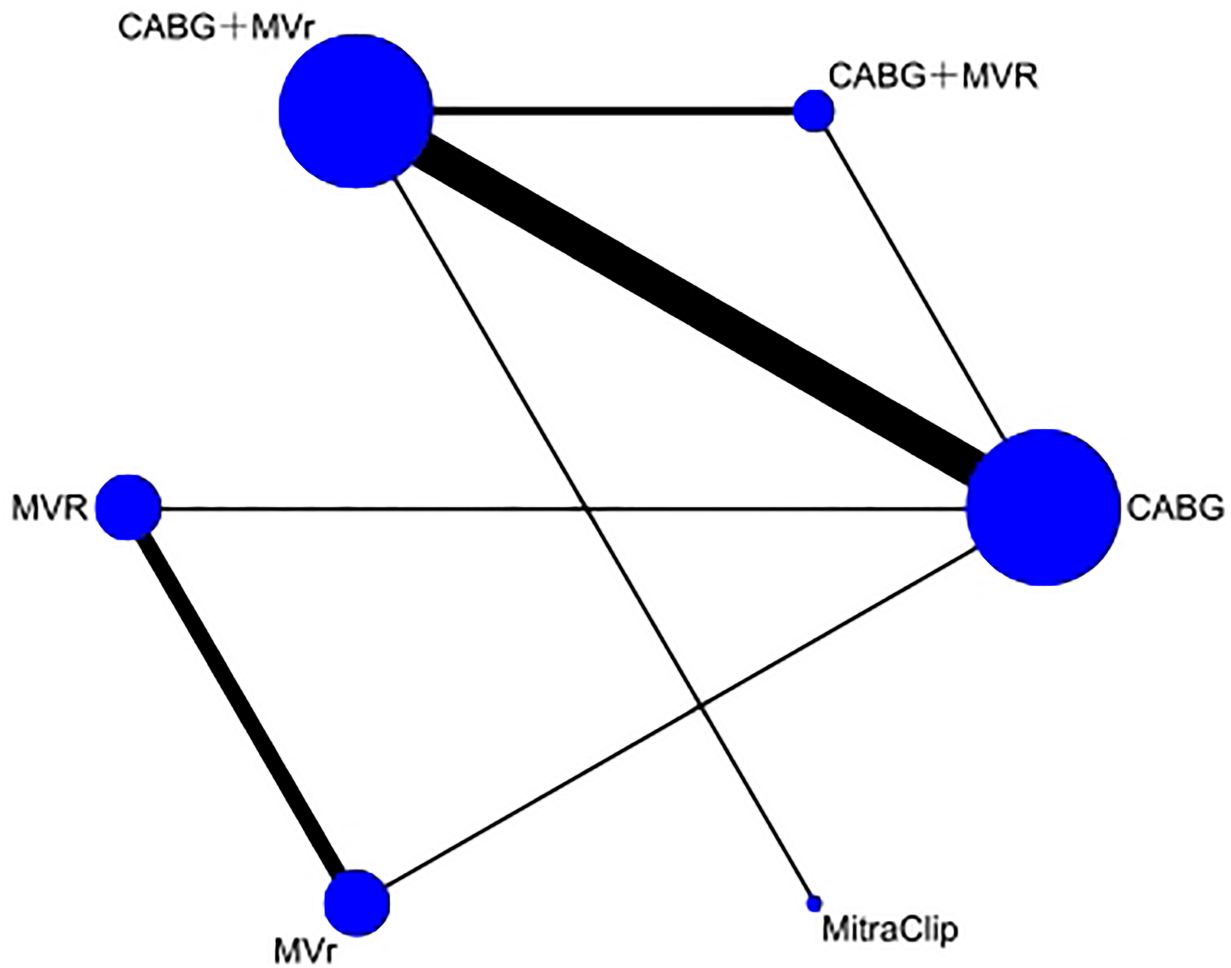
**Network diagram of 30-day all-cause mortality rate**. CABG, coronary artery bypass grafting; MVR, mitral valve replacement; MVr, mitral valve repair.

Focusing on the 30-day all-cause mortality rate, the network diagram is illustrated in Fig. 3. This diagram displays several interventions forming closed network relationships. The most substantial comparison, involving seven experiments, was between CABG and CABG+MVr. Comparisons among other interventions were less frequent in original studies, with CABG+MVr involving the largest participant group (947 participants).

Details of the probability ranking of all outcome measures are provided in Table 3, and the Grade tool was used to evaluate the conclusions obtained from this network meta-analysis, as detailed in Table 4.

**Table 3.** **The probability rankings of all outcomes**.

Intervention	30-day all-cause mortality		Major bleeding events		Stroke		Renal complications		Neurological complications		Respiratory complications	
	SUCRA	Rank	SUCRA	Rank	SUCRA	Rank	SUCRA	Rank	SUCRA	Rank	SUCRA	Rank
CABG+MVr	70.8	2	44.9	3	49.8	3	88.2	1	26.5	4	28.3	3
CABG+MVR	26.0	5	39.4	4	56.0	2	30.0	5	62.8	2	88.4	1
MVr	96.7	1	NR	NR	29.5	4	47.9	3	NR	NR	NR	NR
MVR	60.6	3	NR	NR	23.3	5	15.5	6	NR	NR	NR	NR
CABG	33.4	4	48.3	2	91.3	1	76.5	2	70.7	1	55.4	2
MitraClip	12.5	6	67.4	1	NR	NR	41.9	4	40.0	3	27.9	4

CABG, coronary artery bypass grafting; MVR, mitral valve replacement; MVr, mitral valve repair; SUCRA, the surface under the cumulative ranking curve; NR, not reported.

**Table 4.** **Quality assessment of the Grade tool for each outcome indicator**.

Certainty assessment							№ of patients	Certainty
№ of studies	Study design	Risk of bias	Inconsistency	Indirectness	Imprecision	Publication bias		
30-day all-cause mortality								
20	RCT	serious	Not serious	Not serious	Not serious	Undetected	3111	Moderate
Major bleeding events								
7	RCT	Very serious	Not serious	Not serious	Not serious	Undetected	1226	Low
Stroke								
8	RCT	Not serious	Not serious	Not serious	serious	Undetected	1635	Moderate
Renal complications								
14	RCT	Not serious	Not serious	Not serious	Not serious	Undetected	2388	High
Neurological complications								
5	RCT	Not serious	Not serious	Not serious	Not serious	Undetected	735	High
Respiratory complications								
4	RCT	Not serious	Not serious	Not serious	serious	Undetected	645	Moderate

RCT, randomized controlled trial.

### 3.6 Network Meta-Analysis Results

#### 3.6.1 30-Day All-Cause Mortality Rate

**Fig. 4.**
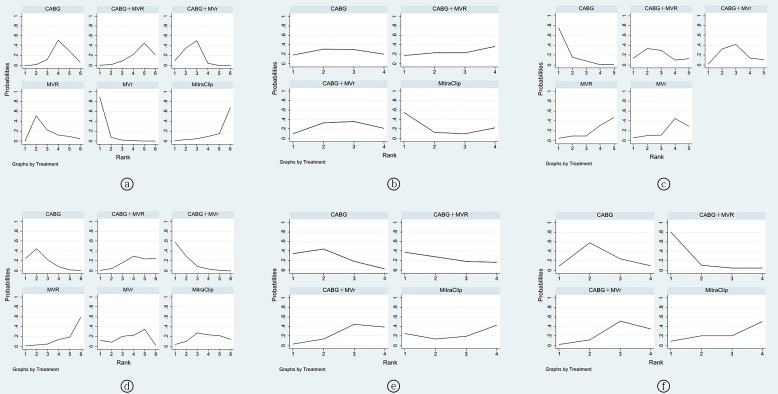
**The probability ranking of all outcomes**. (a) 30-day all-cause mortality rate. (b) Major bleeding events. (c) Stroke. (d) Renal complications. (e) Neurological complications. (f) Respiratory complications. CABG, coronary artery bypass grafting; MVR, mitral valve replacement; MVr, mitral valve repair.

The analysis incorporated data from 20 RCTs [16, 17, 18, 19, 20, 21, 22, 23, 24, 25, 26, 27, 28, 29, 30, 31, 32, 33, 34, 35] focusing on the 30-day all-cause mortality rate. The findings revealed that MVr significantly reduced the 30-day all-cause mortality rate compared to TEER using MitraClip [OR= 8.17, 95% CI (1.20, 55.67)], MVR [OR = 0.43, 95% CI (0.23, 0.79)], CABG+MVR [OR = 0.20, 95% CI (0.04, 0.91)], and CABG [OR = 0.24, 95% CI (0.07, 0.87)]. Furthermore, the 30-day mortality rate for CABG+MVr was significantly lower than for CABG+MVR [OR = 0.48, 95% CI (0.24, 0.94)] and CABG [OR = 0.58, 95% CI (0.35, 0.96)], all with statistical significance (*p* < 0.05), as shown in Table 5. The cumulative probability ranking indicated the following order of effectiveness: MVr > CABG+MVr > MVR > CABG > CABG+MVR > TEER using MitraClip, as depicted in Fig. 4a.

**Table 5.** **The network meta-analysis for all interventions**.

30-day all-cause mortality					
MitraClip					
8.17 (1.20, 55.67)	MVr				
3.51 (0.50, 24.49)	0.43 (0.23, 0.79)	MVR			
3.39 (0.89, 12.88)	0.41 (0.10, 1.64)	0.96 (0.24, 3.94)	CABG+MVr		
1.62 (0.36, 7.24)	0.20 (0.04, 0.91)	0.46 (0.10, 2.17)	0.48 (0.24, 0.94)	CABG+MVR	
1.96 (0.47, 8.18)	0.24 (0.07, 0.87)	0.56 (0.15, 2.08)	0.58 (0.35, 0.96)	1.21 (0.53, 2.77)	CABG
Major bleeding events					
MitraClip					
0.49 (0.03, 9.68)	CABG+MVr				
0.43 (0.01, 14.68)	0.88 (0.13, 5.77)	CABG+MVR			
Stroke					
MVr					
0.89 (0.36, 2.23)	MVR				
3.02 (0.13, 68.84)	3.38 (0.14, 80.38)	CABG+MVr			
3.36 (0.11, 99.18)	3.76 (0.12, 115.45)	1.11 (0.30, 4.08)	CABG+MVR		
8.28 (0.44, 154.17)	9.28 (0.48, 180.52	2.74 (0.91, 8.30)	2.47 (0.45, 13.62)	CABG	
Renal complications					
MitraClip					
0.97 (0.19, 4.86)	MVr				
0.59 (0.11, 3.09)	0.61 (0.34, 1.09)	MVR			
1.96 (0.87, 4.41)	2.03 (0.50, 8.20)	3.33 (0.78, 9.16)	CABG+MVr		
0.83 (0.29, 2.38)	0.85 (0.18, 3.98)	1.40 (0.29, 6.85)	0.42 (0.21, 0.83)	CABG+MVR	
1.68 (0.60, 4.70)	1.73 (0.50, 6.01)	2.85 (0.78, 9.44)	0.85 (0.45, 1.62)	2.03 (0.82, 5.03)	CABG
Neurological complications					
MitraClip					
0.43 (0.01, 13.28)	CABG+MVr				
0.38 (0.09, 1.57)	0.89 (0.04, 9.40)	CABG+MVR			
0.91 (0.12, 6.85)	2.13 (0.04, 9.58)	2.39 (0.20, 9.30)	CABG		
Respiratory complications					
MitraClip					
0.68 (0.17, 2.65)	CABG+MVr				
0.74 (0.37, 1.49)	1.09 (0.34, 3.52)	CABG+MVR			
2.29 (0.52, 10.21)	3.38 (0.45, 9.54)	3.10 (0.60, 9.19)	CABG		

CABG, coronary artery bypass grafting; MVR, mitral valve replacement; MVr, mitral valve repair.

#### 3.6.2 Major Bleeding Events

7 RCTs [17, 18, 24, 29, 31, 33, 35] were included in the evaluation of major bleeding events. The network meta-analysis indicated no significant statistical differences among the various interventions (*p* > 0.05), as outlined in Table 5. The optimal probability ranking for major bleeding events was as follows: TEER using MitraClip > CABG > CABG+MVr > CABG+MVR, as shown in Fig. 4b.

#### 3.6.3 Stroke

8 RCTs [16, 17, 18, 24, 27, 28, 29, 32] were included in the analysis of stroke incidence. The results indicated no significant differences among the interventions (*p* > 0.05), as reported in Table 5. The probability ranking for the likelihood of stroke was as follows: CABG > CABG+MVR > CABG+MVr > MVr > MVR, as presented in Fig. 4c.

#### 3.6.4 Renal Complications

14 RCTs [16, 17, 18, 20, 23, 26, 27, 28, 29, 30, 31, 32, 33, 35] were included in the assessment of renal complications. The network meta-analysis indicated that CABG combined with MVr significantly reduced the incidence of renal complications compared to CABG combined with MVR [OR = 0.42, 95% CI (0.21, 0.83)], with statistical significance (*p* < 0.05). There were no significant statistical differences in renal complications among the remaining interventions (*p* > 0.05), as detailed in Table 5. The probability rankings for the interventions were as follows: CABG+MVr > CABG > MVr > MitraClip > CABG+MVR > MVR, as depicted in Fig. 4d.

#### 3.6.5 Neurological Complications

Neurological complications were assessed in 5 RCTs [23, 29, 32, 34, 35]. The network meta-analysis revealed no significant differences among the various interventions (*p* > 0.05), as presented in Table 5. The probability rankings for the likelihood of neurological complications were as follows: CABG > CABG+MVR > TEER with MitraClip > CABG+MVr, as shown in Fig. 4e.

#### 3.6.6 Respiratory Complications

Respiratory complications were examined in 4 RCTs [23, 29, 31, 35]. The network meta-analysis did not identify significant differences among the interventions (*p* > 0.05), as outlined in Table 5. The probability ranking for respiratory complications suggested the following sequence: CABG+MVR > CABG > CABG+MVr > TEER with MitraClip, as illustrated in Fig. 4f.

## 4. Discussion

As the global population ages, there has been a notable increase in the incidence of Coronary Artery Disease (CAD), a trend attributed to medical advancements that have improved the survival rates of CAD patients. Consequently, there has been a corresponding rise in cases of IMR following CAD surgery [3, 36]. IMR develops as a consequence of myocardial injury induced by CAD, leading to adverse ventricular remodeling. The primary pathological processes of IMR include left ventricle and mitral annulus enlargement, dysfunction, and lateral displacement of the papillary muscles, as well as reduced valvular coaptation. These pathological processes dynamically change with variations in cardiac preload, rhythm, and other factors [37]. Given that the pathology of IMR primarily affects the myocardium rather than the valve itself, its treatment significantly differs from that of degenerative MR. The limitation of solely performing CABG is the potential for residual and recurrent MR. Conversely, procedures like mitral valve repair or replacement, as well as other related surgical procedures, have drawbacks such as longer cardiopulmonary bypass times and inadequate revascularization of the ischemic myocardium. Interventional treatment for IMR is still in its exploratory stages, thus determining the specific efficacies of various surgical treatments for IMR is a pressing concern.

A 2009 study by Fattouch* et al*. [38] found no statistical difference in the 30-day all-cause mortality rate between IMR patients undergoing only CABG and those undergoing CABG combined with MVr (OR = 1.58, *p *= 0.29). In 2020, research by Dufendach *et al*. [18] showed that the 30-day all-cause mortality rate for IMR patients undergoing CABG combined with MVR was notably higher than for CABG combined with MVr (OR = 1.95, *p* = 0.04), and the incidence of renal complications was also higher in the CABG combined with MVR group (OR = 2.38, *p* < 0.01). A 2014 study by Acker *et al*. [16] reported no significant differences in the 30-day all-cause mortality rate (OR = 0.41, *p* = 0.26) and stroke (OR = 0.58, *p* = 0.97) between MVR and MVr in IMR patients. A 2017 study by Qiu *et al*. [30] also found no statistical difference in the 30-day all-cause mortality rate between MVR and MVr (OR = 0.56, *p* = 0.7). In a 2023 study, Andrási *et al*. [39] discovered that IMR patients undergoing CABG with MVr exhibited a lower postoperative mortality rate compared to those undergoing CABG with MVR. These findings reveal inconsistent conclusions from previous comparisons of surgical methods in IMR patients, leading to our network meta-analysis of the efficacies of different surgical treatments for IMR. The network meta-analysis results show no statistical differences in major bleeding events, stroke, neurological, and respiratory complications among the surgical methods, aligning with previous studies [17, 23]. This indicates that the choice of surgical approach does not affect coagulation, neurological, or respiratory functions in patients. In terms of 30-day all-cause mortality, MVr significantly outperformed TEER with MitraClip, MVR, CABG+MVR, and CABG, and CABG+MVr was significantly more effective than CABG and CABG+MVR. MVr was found to be the most effective in reducing 30-day all-cause mortality, consistent with previous research [20, 22, 26]. In the context of renal complications, CABG+MVr showed significantly fewer complications compared to CABG+MVR, with MVR being most effective in lowering the incidence of renal complications, indicating minimal renal damage from MVR. The probable causes for these differences are as follows: (1) CABG alone cannot reverse myocardial scarring and fibrosis following ischemia, thus ineffectively reversing myocardial remodeling and improving cardiac function [40, 41]; (2) MVr or CABG+MVr involves shorter surgery time, reducing the duration of cardiopulmonary bypass and aortic clamping, thus causing less organ damage and significantly lowering complication rates and short-term postoperative mortality [42]; (3) Compared to MVr, CABG alone often leads to residual and recurrent MR, which can result in chronic heart failure and reduced survival rates [43].

### Limitations

This research encompasses certain limitations: (1) The type of valve used in patients undergoing MVR, MVr, or associated surgeries differs, which might have an impact on the outcomes; (2) The preoperative cardiac functions of patients are varied, leading to potential disparities in post-surgery mortality and the occurrence of various complications; (3) The majority of included studies have relatively small sample sizes, raising the possibility of Type II errors; (4) The study lacks a detailed description and differentiation of the grades of MR; (5) This study only included RCTs and excluded retrospective studies, which may introduce bias into the research findings.

## 5. Conclusions

In conclusion, based on the findings of this study, MVr or CABG+MVr seems to be the most effective approach in reducing short-term mortality for IMR patients. When compared to CABG+MVR, CABG+MVr leads to less damage to renal function. Additionally, no notable differences were observed in major bleeding events and other complications among the different surgical treatments for IMR. However, it is important to note that these conclusions require further validation through more stringent clinical trials.

## Availability of Data and Materials

The original data has been uploaded as an attachment to the manuscript.

## Supplementary Material

Supplementary material associated with this article can be found, in the online version, at https://doi.org/10.31083/j.rcm2511425.
